# Effect of Deep Sedation on Mechanical Power in Moderate to Severe Acute Respiratory Distress Syndrome: A Prospective Self-Control Study

**DOI:** 10.1155/2020/2729354

**Published:** 2020-04-11

**Authors:** Yongpeng Xie, Lijuan Cao, Ying Qian, Hui Zheng, Kexi Liu, Xiaomin Li

**Affiliations:** ^1^Department of Critical Care Medicine, Lianyungang Clinical College of Nanjing Medical University, The First People's Hospital of Lianyungang City, Lianyungang, China; ^2^Department of Emergency Medicine, Lianyungang Clinical College of Nanjing Medical University, The First People's Hospital of Lianyungang City, Lianyungang, China

## Abstract

Mechanical power (MP) is a parameter for assessing ventilator-induced lung injury (VILI) in patients with acute respiratory distress syndrome (ARDS). Deep sedation inhibits the respiratory center and reduces the excessive spontaneous breathing in ARDS patients, thereby reducing transpulmonary pressure (Ptp) and lung injury. However, the effect of sedation on MP in ARDS patients is not yet clear. Therefore, the purpose of this study was to investigate the effect of deep sedation on MP in ARDS patients. Patients with moderate to severe ARDS who required mechanical ventilation were considered. Different degrees of sedation were performed on patients in three stages after 24 hours of mechanical ventilation. The three stages are as follows: stage 1 (H+3): 0 to 3 hours of sedation; patients' Ramsay score was 2-3 to obtain mild sedation; stage 2 (H+6): 4 to 6 hours of sedation; the sedation depth was adjusted to 5-6 points; and stage 3 (H+9): 7 to 9 hours of sedation; the sedation depth was adjusted to 2-3 points. Under deep sedation (H+6), MP, respiratory rate (RR), and Ptp were significantly lower than the ones in the patients under mild sedation (H+3) (all *P* < 0.01) although PaO_2_*/F*iO_2_ (P/F) and static lung compliance (Cst) were significantly higher (both *P* < 0.01). However, no significant difference in the above parameters was observed between H+3 and H+9. Correlation analysis showed that *Δ*MP was significantly and positively correlated with *Δ*RR and *Δ*Ptp (both *P* < 0.001), while no correlation was observed neither between *Δ*MP and *Δ*Cst nor between *Δ*MP and *Δ*P/F. The 28-day Kaplan-Meier survival curve showed the occurrence of 19 deaths, and the overall survival rate was 63.46%. The survival rate was 53.12% in the high-MP (HMP) group and 80.95 in the low-MP (LMP) group (*P* < 0.05). In conclusion, deep sedation significantly reduced MP in patients with moderate to severe ARDS, thereby reducing the occurrence of VILI. In addition, MP monitoring in deep sedation predicted the 28-day survival of patients with moderate to severe ARDS.

## 1. Introduction

The acute respiratory distress syndrome (ARDS) is a common disease in critical care medicine, accounting for 10% of intensive care unit patients, and the mortality rate is as high as 46% [1]. Mechanical ventilation plays a key role in the treatment of patients with ARDS, although it may cause ventilator-induced lung injury (VILI) [2]. In recent years, it has been found that, during mechanical ventilation of ARDS patients, mechanical power (MP) can be used to evaluate and prevent VILI compared to other mechanical parameters, such as driving pressure, transpulmonary pressure (Ptp), respiratory rate (RR), and positive end-expiratory pressure (PEEP) [3]. It indicates the energy that the ventilator delivers to the entire respiratory system in one minute, expressed as J/min. MP embodies the weight calculation of all respiratory mechanical parameters that can cause VILI. At present, MP is confirmed to be an effective method for assessing and preventing VILI [4]. In the future, MP may become the new standard to perform lung-protective ventilation in ARDS patients [5]. Studies have shown that when MP exceeds 17 J/min, the 28-day mortality in patients increases significantly, which indicated that the level of MP is related to the prognosis of patients and may be related to the severity of ARDS [6, 7]. Whether spontaneous respiration in ARDS is helpful or harmful remains an area of debate [8]. Clinical studies confirmed that sedation inhibits the driving of the respiratory center and reduces the amplitude of spontaneous respiration, especially for early moderate to severe ARDS, deep sedation and even muscle relaxation can greatly improve the prognosis of patients and reduce the incidence of VILI [9, 10]. However, for mild ARDS or convalescent ARDS, it is recommended to keep spontaneous respiration, which may be more conducive to the recovery of the disease. Thus, our hypothesis is that deep sedation may reduce MP by inhibiting spontaneous breathing in early moderate to severe ARDS, thereby preventing VILI. However, no clinical study is available on the effect of sedation on MP in patients with ARDS. Therefore, in this study, the differences of MP in patients with ARDS at different sedation depths during volume-controlled ventilation were investigated to evaluate the effect of sedation on MP in patients with ARDS.

## 2. Materials and Methods

### 2.1. Patients

Patients with moderate to severe ARDS, who required mechanical ventilation therapy longer than 36 hours, were randomly selected among hospitalized patients from June 2017 to June 2019. The inclusion criteria were as follows: age ≥ 18 years; compliance with the 2012 ARDS Berlin definition of moderate to severe ARDS diagnostic criteria, with a PaO_2_/*F*iO_2_ (P/F) ≤ 200 mmHg [11]; invasive mechanical ventilation treatment; and estimated mechanical ventilation treatment for more than 36 hours. The exclusion criteria were as follows: age < 8 years, pregnancy, esophageal obstruction, esophageal perforation, severe esophageal varix bleeding, upper gastrointestinal tract and transthoracic surgery, thoracic deformity, pneumothorax, massive pleural effusion, history of pulmonary alveolar disease, severe hemodynamic abnormalities, severe heart failure, and acute coronary syndrome. The elimination criteria were as follows: the primary disease was aggravated or repeated; RR > 40 bpm or ≤8 bpm; SPO_2_ < 88%; blood pressure fluctuation greater than 30 mmHg or malignant arrhythmia; patients with abnormal irritability that compromised the participation to this study; and the clinician was forced to finish the study due to the patient's health condition. During a 24-month period, 93 patients with moderate to severe ARDS were originally screened. 41 patients were excluded because they qualified for the exclusion criteria, including 11 patients with severe hemodynamic abnormalities, 5 patients with pulmonary alveolar disease, 6 patients with upper gastrointestinal tract or transthoracic surgery, 2 patients with thoracic deformity, 5 patients with massive pleural effusion, 9 patients with acute and chronic heart failure, and 3 patients who were younger than 18 years. Finally, 52 patients were included in this study.

### 2.2. Ethics Statement

The research was conducted through the medical research registration system of the National Health Commission of China, and the protocol was approved by the ethics committee of the Lianyungang Clinical College of Nanjing Medical University, with the approval number LCYJ20170312001. Before the beginning of the study, a written informed consent was obtained from each patient's legal representative. Patient records/information was anonymized and deidentified prior to analysis. The Chinese Clinical Trial Registry number is ChiCTR1900028238

### 2.3. Mechanical Ventilation

All the included patients were ventilated according to the original ARDSnet protocol [12]. Briefly, patients were ventilated in a volume-assisted control mode with a constant square flow and a tidal volume of 6 ml/kg/IPBW (ideal predicted body weight) using the PB840 ventilator (Tyco Healthcare, USA). There would be a short pause to obtain the plateau pressure. The goal of oxygenation was to target a peripheral saturation of blood oxygen measured by pulse oximetry between 88 and 95% or a PaO_2_ of 55–80 mmHg measured by arterial blood gas analysis. To achieve this goal, FiO_2_ and PEEP were adjusted according to the table of PEEP and FIO_2_ combinations as in the ARMA and ACURASYS study (Table 1) [13]. RR was adjusted to ensure an arterial pH between 7.20 and 7.45.

### 2.4. MP Monitoring in ARDS Patients

The ventilator monitors the following respiratory mechanical parameters including tidal volume (VT), peak pressure (Ppeak), RR, platform pressure (Pplat), and positive end-expiratory pressure (PEEP). The driving pressure (DP) was calculated using the following formula: DP = Pplat − PEEP. MP was calculated as previously described using VT, Ppeak, RR, and DP as follows: MP (J/min) = 0.098 × VT × RR × (Ppeak − 1/2 × DP) [7]. This study started after 24 hours of mechanical ventilation. Patient's MP was recorded at three different sedation treatment stages (H+3, H+6, and H+9). The mean of the three measured MP at each stage was used. Since a consistent increase in the risk of death was observed with MP higher than 17.0 J/min [7], patients were divided into a high-MP group (HMP) and low-MP group (LMP) depending on whether MP was higher or lower than 17.0 J/min. Next, patients' 28-day survival status was analyzed and recorded.

### 2.5. Esophageal and Ptp Measurement

A standard procedure was followed to place the manometry catheter [14]. The esophageal catheter reached the throat through the nasal cavity. With the swallowing activity, the catheter enters the esophagus and is placed at a depth of 60 cm to reach the stomach. Five ml of gas was inflated into the esophageal balloon, and then, 4 ml of the gas was pumped back. The esophageal catheter was connected with the esophageal pressure sensor, thus instantly monitoring the actual intragastric pressure, and the pressure-time waveform was constantly positive. The catheter was slowly withdrawn outward and returned to the thoracic esophagus to evaluate if the esophageal pressure-time waveform became a sine wave with the respiratory motion. The esophageal catheter position was confirmed by the “occlusion test”: after blocking the exhalation, the esophageal tube position was considered correct when the patient's airway pressure change value (*Δ*Paw) and the esophageal pressure change value (*Δ*Pes) were basically the same as the patient's efforts to inhale and exhale, respectively [15]. The esophageal pressure (Pes) was monitored to indirectly reflect intrathoracic pressure; inspiratory Ptp was the difference between the alveolar pressure and the inspiratory intrathoracic pressure (Pesinsp). The inspiratory Ptp was calculated as follows: Ptp = Pplat − Pesinsp[16].

### 2.6. Management of Sedation

**Figure 1:**
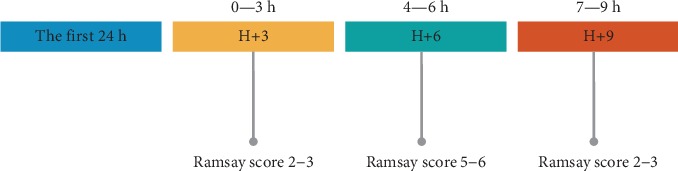
The time stamp for each sedation stage.

During the first 24 h of ventilation, the Ramsay sedation scale was used to adapt the sedative requirements. The scale assigns a score from 1 (conscious state: anxious, agitated, or restless) to 6 (no response on glabellar tap) [17]. A continuous infusion of propofol (0.3-2.0 mg/kg·h) and dexmedetomidine (0.2-1.0 *μ*g/kg·h) was used to achieve a Ramsay score of 6 throughout the study. If this goal was not achieved, a continuous infusion of midazolam (0.04-0.15 mg/kg·h) was added. All patients were given remifentanil (2.0-3.0 *μ*g/kg·h) for analgesia during mechanical ventilation. This study started from the second 24 hours; the depth of sedation was modified by adjusting the amount of drug infusion. There are three stages as follows: stage 1 (H+3): 0 to 3 hours of sedation; the sedation depth was adjusted to a Ramsay score of 2-3 to obtain mild sedation; stage 2 (H+6): 4 to 6 hours of sedation; the sedation depth was adjusted to a score of 5-6; and stage 3 (H+9): 7 to 9 hours of sedation; the sedation depth was adjusted to a score of 2-3 (Figure 1).

### 2.7. Statistical Analysis

Data processing and mapping were performed using the SPSS 22.0 statistical software and GraphPad Prism 6.0. Results were expressed as mean ± standard deviation (*x* ± *s*) of at least three different measurements. Differences between two groups were compared using two independent-sample *t*-tests. The correlation between *Δ*MP and *Δ*RR, *Δ*Ptp, *Δ*Cst, and *Δ*PF was analyzed by Spearman's correlation analysis. The 28-day mortality of the patients was recorded, and the predictive MP value on the 28-day survival status of patients was assessed by the Kaplan-Meier survival curves. *P* < 0.05 was considered statistically significant.

## 3. Results

### 3.1. Characteristics of the Patients with ARDS

The characteristics of the patients included in this study are listed in Table 2.

### 3.2. MP, RR, Ptp, Cst, and PF at Different Sedation Depths in ARDS Patients

**Figure 2:**
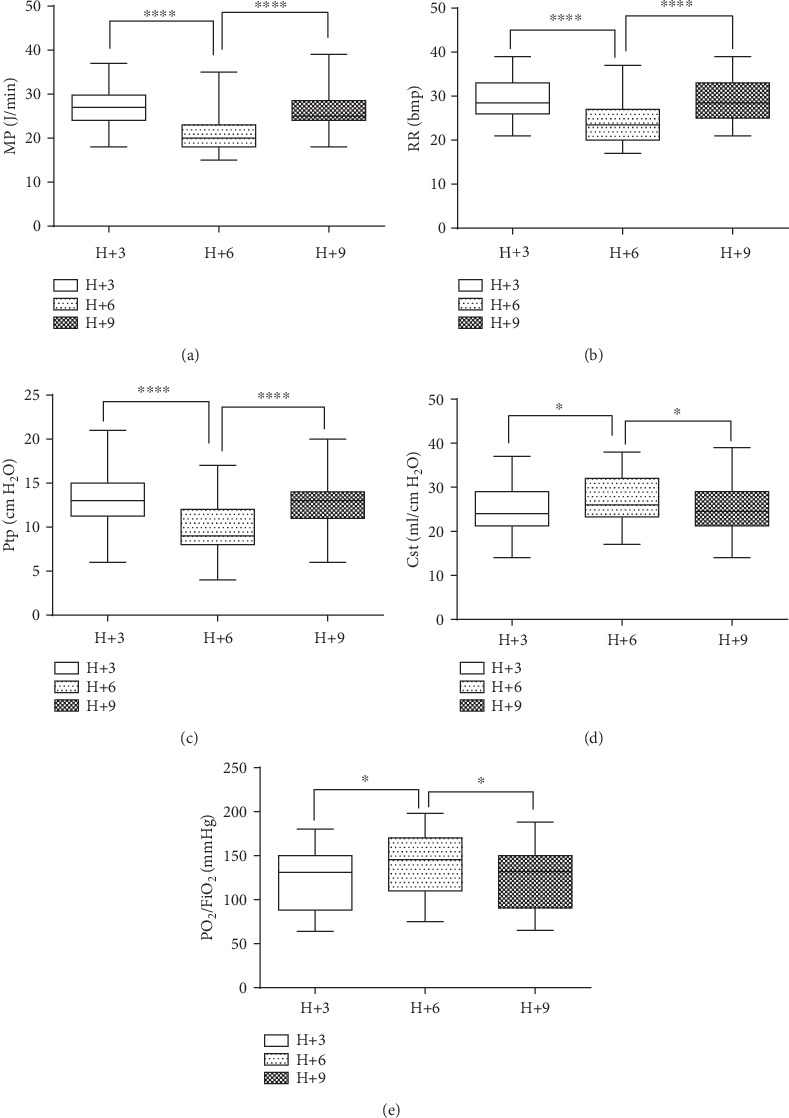
Parameter variation in ARDS patients under moderate to severe sedation. (a) MP. (b) RR. (c) Ptp. (d) Cst. (e) PF. Results were expressed as mean ± standard deviation (*x* ± *s*) of at least three different measurements. ^∗^*P* < 0.05, ^∗∗∗∗^*P* < 0.0001.

Changes in respiratory mechanics and oxygenation in different stages of sedation are illustrated in Figure 2. Under deep sedation (H+6), MP, RR, and Ptp were significantly lower than the ones in the patients under mild sedation (H+3) [MP (J/min): 20.59 ± 3.80*vs.*26.52 ± 4.49; RR (bmp): 21.73 ± 3.31*vs.*27.71 ± 4.78; Ptp (cm H_2_O) 10.25 ± 3.42*vs.*13.75 ± 3.62; all *P* < 0.01], although P/F and Cst were significantly higher [P/F (mmHg): 142.46 ± 33.76*vs.*121.52 ± 35.89, Cst (ml/cm H_2_O) 27.3 ± 4.62*vs.*24.62 ± 5.25; both *P* < 0.01]. However, no significant difference in the above parameters was observed between the two mild sedation stages, H+3 and H+9.

### 3.3. Correlation between *Δ*MP and *Δ*RR, *Δ*Ptp, *Δ*Cst, and *Δ*PF in Patients with ARDS

**Figure 3:**
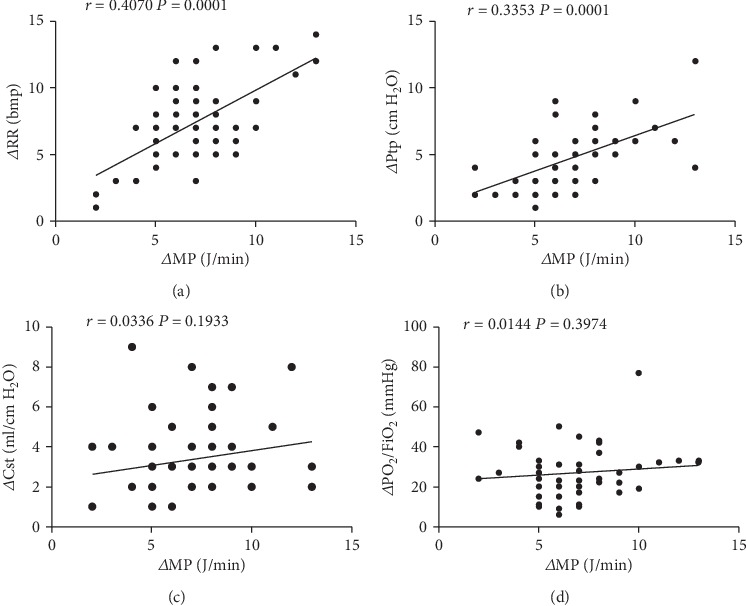
Correlation between *Δ*MP and *Δ*RR, *Δ*Ptp, *Δ*Cst, and *Δ*PF. (a) *Δ*MP *vs. Δ*RR. (b) *Δ*MP *vs. Δ*Ptp. (c) ΔMP *vs. vs. Δ*Cst. (d) *Δ*MP *vs. Δ*PF. Results were expressed as mean ± standard deviation (*x* ± *s*) of at least three different measurements.

The variation in MP, RR, Ptp, Cst, and PF between different sedation depths (the 3rd hour and 6th hour) was calculated and recorded as *Δ*MP, *Δ*RR, *Δ*Ptp, *Δ*Cst, and *Δ*PF, respectively. The correlation analysis showed that *Δ*MP was significantly and positively correlated with *Δ*RR and *Δ*Ptp (*r* values 0.4070 and 0.3353, respectively; both *P* < 0.001), while no correlation was observed neither between *Δ*MP and *Δ*Cst nor between *Δ*MP and *Δ*P/F (*r* values 0.0336 and 0.0144, respectively) (Figure 3).

### 3.4. Predictive MP Value for 28-Day Survival Status in Patients with ARDS

**Figure 4:**
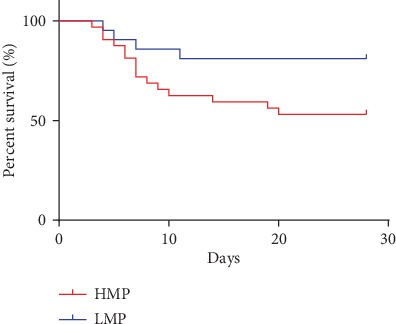
MP and 28-day survival curve in patients with ARDS.

The 28-day survival status of the enrolled patients was assessed, and all patients were divided into the HMP group (32) and LMP group (20). The 28-day Kaplan-Meier survival curve showed the occurrence of 19 deaths, and the overall survival rate was 63.46%. The 28-day survival rate in the HMP group was 53.12%, while that in the LMP group was 75.00%, and the difference was statistically significant (*P* < 0.05) (Figure 4). In addition, 17 cases (85.00%) were successfully extubated in the LMP group and 21 cases (65.62%) in the HMP group. There was no significant statistical difference (*P* > 0.05).

## 4. Discussion

Mechanical ventilation is still the most important supportive treatment in patients with ARDS. Lung ventilation is the dynamic process of breathing to drive gases in and out the lungs [18]. During the mechanical ventilation treatment in ARDS, improper ventilator settings or excessive spontaneous breathing can cause ventilator-related damage, aggravate disease progression, and increase mortality [19]. If the mechanical damage to the lung parenchyma is a function of the MP, it is possible that different combinations of its components resulting in a MP greater than a given threshold may produce similar damages, as recently suggested by animal experiments [20]. Sedative treatment can significantly affect the patient's respiratory mechanical parameters including RR, Ptp, and air resistance [21]. Therefore, in this study, the effect of different depths of sedation on MP was evaluated in patients with moderate to severe ARDS, demonstrating that sedation treatment has an important clinical value in preventing the occurrence of VILI in patients with ARDS.

This study showed that MP in patients with ARDS with a sedation depth at Ramsay 5 was significantly lower than that in patients at Ramsay 3, suggesting that deep sedation reduced MP in patients with ARDS. In addition, deep sedation significantly reduced the patient's RR, and, at different depths of sedation, the variations in MP and RR showed a significant positive correlation. And our previous study [22] proved that there is a positive correlation between MP and RR and Ptp and a negative correlation with lung compliance. It is consistent with the conclusions of Gattinoni et al.'s research which suggested that RR has a significant effect on MP in patients with moderate to severe ARDS, since patients' RR on MP can reach 27% [5]. Furthermore, a higher MP inevitably leads to more serious lung damage caused by the ventilator. This is consistent with the results of animal studies by Cressoni et al. [4]. They increased MP by continuously increasing the RR of healthy pigs, resulting in a more severe VILI in healthy pigs with high RR. Therefore, this study confirmed in vivo in patients that excessive RR during mechanical ventilation in patients with moderate to severe ARDS also caused VILI by increasing MP, consequently increasing mortality.

Our results also demonstrated that deep sedation significantly reduced the inspiratory Ptp in patients with ARDS and showed a significant positive correlation with the reduction in MP. These results suggested that the patient's spontaneous breathing amplitude was significantly reduced after deep sedation, and the deeper the sedation, the more remarkable the inhibition of the patient's respiratory center drive [23]. Thus, the diaphragmatic contraction function was also reduced, significantly reducing the level of inspiratory Ptp [24]. A study showed that a significant increase in Ptp is also one of the important causes of lung injury [25]. In patients with ARDS who are subjected to mechanical ventilation, even if their pressure support is not high, when they make strong spontaneous breathing efforts, the Ptp may increase significantly due to the significant decrease in intrathoracic pressure, which may aggravate lung injury [26]. Therefore, the inhibition of the spontaneous breathing efforts in ARDS patients by deep sedation significantly reduced the MP, at the same time significantly reducing the inspiratory Ptp. This may be important to reduce the occurrence of VILI.

The study also found that patients' oxygenation index and lung compliance improved in the deep sedation phase compared to the mild sedation phase, suggesting that deep sedation improved the oxygenation status and lung compliance in patients with ARDS. However, there is no clear correlation between *Δ*MP and *Δ*Cst or *Δ*MP and *Δ*P/F. The reason could be due to the fact that the patient's oxygenation status was affected by more factors, including oxygen concentration level, lung ventilation, and lung gas exchange function [27]. The lung compliance is affected by the severity of pulmonary edema, pulmonary surfactant content, airway resistance, pleural effusion, and anesthetic sedatives [28]. This study also showed that deep sedation mainly reduced MP and Ptp by inhibiting the patient's excessive spontaneous breathing frequency, without significant correlation with changes in airway resistance and tidal volume, gas flow rate, and lung compliance. During the treatment of ARDS, we also need to emphasize the timely adjustment of the depth of sedation and the amount of sedative drugs according to the different conditions of the patient. When the patient's condition improves, we must avoid deep sedation and even do not recommend sedative. The aim is to reduce prolonged ventilator use and ventilator dependence caused by excessive sedation.

In this study, patients were divided into the high-MP group and low-MP group depending on whether MP in the deep sedation stage exceeded 17.0 J/min or not. The survival rate of the two groups was significantly different, indicating that the MP level was clearly related to the prognosis of the patients, and it was an independent risk factor for the death of the patients. Moreover, it suggested that the severity of the disease can be judged according to the difference of the patient's MP. The higher MP indicates the heavier lung injury of the ARDS patients [22], because patients in the high-MP group may have more severe alveolar collapse, atelectasis, pulmonary edema, and even lung consolidation. In order to open the collapsed alveoli and maintain them open, the ventilator should exert more power during the mechanical ventilation of ARDS patients [29], meaning that more MP is needed. Therefore, MP is closely related to the severity of lung damage in patients with ARDS and can be used to assess the prognosis of patients.

## 5. Limitations

First, this study was a single-center trial with a small number of cases. Thus, further multicenter and large-sample clinical studies are needed to confirm the effects of deep sedation on MP in moderate to severe ARDS. Second, this trial only commenced after 24 hours and not within 24 hours. Because we believe that the classification of ARDS with the oxygenation index after 24 hours may be more accurate, the oxygenation index at this time is the result of intervention by our treatment measures, and it may be more stable than the initial stage of mechanical ventilation. In addition, patients need a large amount of therapeutic intervention within 24 hours, and conducting this study at this time may be detrimental to patients. Third, in order to avoid human bias, we adopted a uniform standard for the setting of ventilator parameters for all patients, especially the setting of respiratory rate. These operations were performed by two researchers together. Finally, the calculation of MP during spontaneous breathing is challenging as airway pressure, flow, and esophageal pressure are affected counterdirectionally and simultaneously overlapping by the action of the ventilator and the respiratory muscles.

## 6. Conclusions

In conclusion, our results demonstrated that MP underlines the usually neglected, but potentially relevant effect of RR, as the power increased exponentially when the RR increased. Deep sedation significantly reduced MP in patients with moderate to severe ARDS, thereby reducing VILI. In addition, MP monitoring under deep sedation could be used to assess prognosis in patients with ARDS.

## Figures and Tables

**Table 1:** PEEP and FIO_2_ combination tables.

Lower PEEP/FIO_2_ combination														
FIO_2_	0.3	0.4	0.4	0.5	0.5	0.6	0.7	0.7	0.7	0.8	0.9	0.9	0.9	1.0
PEEP	5	5	8	8	10	10	10	12	14	14	14	16	18	18-24

Higher PEEP/FIO_2_ combination														
FIO_2_	0.3	0.3	0.4	0.4	0.5	0.5	0.5	0.6	0.7	0.8	0.8	0.9	1.0	
PEEP	12	14	14	16	16	18	20	20	20	20	22	22	22-24	

PEEP: positive end-expiratory pressure (cmH_2_O).

**Table 2:** Characteristics of the patients with ARDS enrolled in this study.

Characteristics (*n* = 52 patients)	Value
Baseline characteristics	
Age (years)	61.35 ± 14.78
Male/total	34/52
Weight (kg)	59.22 ± 11.43
BMI (kg/m^2^)	24.98 ± 3.75
SOFA	8.87 ± 3.76
APACHE II	19.45 ± 6.55
PaO_2_/FiO_2_	130.31 ± 48.87
Moderate ARDS	34/52
Severe ARDS	18/52
Comorbidities	
CAD	6/52
DM	9/52
Hypertension	11/52
Liver cirrhosis	3/52
Causes of ARDS	
Pneumonia	23/52
Sepsis	14/52
Trauma or burn	8/52
SAP	2/52
Drowning	2/52
Others	3/52
Ventilation characteristics	
Tidal volume (ml)	358.45 ± 53.65
PEEP (cm H_2_O)	12.43 ± 4.24
Driving pressure (cm H_2_O)	16.65 ± 3.43
Plateau pressure (cm H_2_O)	24.54 ± 6.53
Minute ventilation (l/min)	7.34 ± 2.23
FiO_2_ (%)	0.63 ± 0.25
Extubation success	38/52
ICU length of stay (days)	9.36 ± 3.25
Hospital length of stay (days)	17.75 ± 6.24

Results were expressed as mean ± standard deviation or number/total. ^∗^BMI: body mass index; SOFA: sequential organ failure assessment; CAD: coronary artery disease; DM: diabetic mellitus; SAP: severe acute pancreatitis; RM: recruitment maneuver; PPV: prone position ventilation; ECMO: extracorporeal membrane oxygenation; PEEP: positive end-expiratory pressure; ICU: intensive care unit.

## Data Availability

The data used to support the findings of this study are available from the corresponding author upon request.
